# Cost-effectiveness of single-visit cervical cancer screening in KwaZulu-Natal, South Africa: a model-based analysis accounting for the HIV epidemic

**DOI:** 10.3389/fonc.2024.1382599

**Published:** 2024-04-24

**Authors:** Jacinda Tran, Christine Lee Hathaway, Cara Jill Broshkevitch, Thesla Palanee-Phillips, Ruanne Vanessa Barnabas, Darcy White Rao, Monisha Sharma

**Affiliations:** ^1^The Comparative Health Outcomes, Policy, and Economics (CHOICE) Institute, Department of Pharmacy, University of Washington, Seattle, WA, United States; ^2^Division of Infectious Diseases, Massachusetts General Hospital, Boston, MA, United States; ^3^Department of Epidemiology, University of North Carolina at Chapel Hill, Chapel Hill, NC, United States; ^4^Faculty of Health Sciences, Wits RHI, University of the Witwatersrand, Johannesburg, South Africa; ^5^Department of Epidemiology, University of Washington, Seattle, WA, United States; ^6^Division of Infectious Diseases, Department of Medicine, Harvard Medical School, Boston, MA, United States; ^7^Department of Global Health, University of Washington, Seattle, WA, United States

**Keywords:** cervical cancer screening, cervical cancer prevention, economic evaluation, human papillomavirus, human immunodeficiency virus

## Abstract

**Introduction:**

Women living with human immunodeficiency virus (WLHIV) face elevated risks of human papillomavirus (HPV) acquisition and cervical cancer (CC). Coverage of CC screening and treatment remains low in low-and-middle-income settings, reflecting resource challenges and loss to follow-up with current strategies. We estimated the health and economic impact of alternative scalable CC screening strategies in KwaZulu-Natal, South Africa, a region with high burden of CC and HIV.

**Methods:**

We parameterized a dynamic compartmental model of HPV and HIV transmission and CC natural history to KwaZulu-Natal. Over 100 years, we simulated the status quo of a multi-visit screening and treatment strategy with cytology and colposcopy triage (South African standard of care) and six single-visit comparator scenarios with varying: 1) screening strategy (HPV DNA testing alone, with genotyping, or with automated visual evaluation triage, a new high-performance technology), 2) screening frequency (once-per-lifetime for all women, or repeated every 5 years for WLHIV and twice for women without HIV), and 3) loss to follow-up for treatment. Using the Ministry of Health perspective, we estimated costs associated with HPV vaccination, screening, and pre-cancer, CC, and HIV treatment. We quantified CC cases, deaths, and disability-adjusted life-years (DALYs) averted for each scenario. We discounted costs (2022 US dollars) and outcomes at 3% annually and calculated incremental cost-effectiveness ratios (ICERs).

**Results:**

We projected 69,294 new CC cases and 43,950 CC-related deaths in the status quo scenario. HPV DNA testing achieved the greatest improvement in health outcomes, averting 9.4% of cases and 9.0% of deaths with one-time screening and 37.1% and 35.1%, respectively, with repeat screening. Compared to the cost of the status quo ($12.79 billion), repeat screening using HPV DNA genotyping had the greatest increase in costs. Repeat screening with HPV DNA testing was the most effective strategy below the willingness to pay threshold (ICER: $3,194/DALY averted). One-time screening with HPV DNA testing was also an efficient strategy (ICER: $1,398/DALY averted).

**Conclusions:**

Repeat single-visit screening with HPV DNA testing was the optimal strategy simulated. Single-visit strategies with increased frequency for WLHIV may be cost-effective in KwaZulu-Natal and similar settings with high HIV and HPV prevalence.

## 1 Introduction

Cervical cancer is the fourth most commonly diagnosed cancer globally, and it disproportionately impacts women in low- and middle-income countries (LMIC) where screening coverage is low. While effective screening strategies are available and have been successfully implemented in high income countries, lack of infrastructure, specialized equipment, and trained health and laboratory personnel remain structural barriers to scale-up in LMICs (1, 2). In 2020, the World Health Organization (WHO) unveiled a worldwide strategy aimed at eradicating cervical cancer and achieving the ambitious 90-70-90 targets by 2030 which encompass: fully vaccinating 90% of girls against HPV by 15 years old, screening 70% of women twice with high performance tests by age 35 and 45 years old, and treating 90% of women with pre-cancerous lesions or cervical cancer (3).

South Africa has one of the highest cervical cancer incidence and mortality rates globally, with over 10,000 new cervical cancer cases and nearly 6,000 cervical cancer-related deaths in 2020 (4). The South African province of KwaZulu-Natal stands as a microcosm of these broader global health challenges, with a disproportionate burden of cervical cancer and high prevalence rates of human immunodeficiency virus (HIV). These two public health issues converge in this region because women living with HIV (WLHIV) are at increased risk of acquiring human papillomavirus (HPV), the primary cause of cervical cancer, and their HPV infections are more likely to progress to cancer (5–8).

With approximately 4.8 million WLHIV in South Africa as of 2022, the burden of HPV and cervical cancer is high, despite high coverage of antiretroviral therapy (7, 9, 10). In 2017, HIV prevalence in KwaZulu Natal was estimated to be 37%, reaching a peak of 59% among women 30 to 49 years old (11–13). The region has historically high HPV prevalence, with estimates 2.5 times higher in WLHIV compared to women without HIV (14). Stelzle et al. estimated that 63.4% of new cervical cancer cases in South Africa were WLHIV in 2018 (5), highlighting the impact of HIV on cervical cancer incidence. Although recent data suggest a decline in HIV incidence in KwaZulu-Natal (12, 13, 15), cervical cancer incidence continues to rise (16), emphasizing the need for greater cervical cancer prevention and screening, particularly among WLHIV.

Coverage of cervical cancer prevention programs in South Africa remains low, reflecting challenges with resource allocation for screening, diagnosis, and access to adequate care (17, 18), in addition to individual and societal barriers such as lack of awareness and misconceptions of cervical cancer (19–21). Barriers to effective scale-up persist at each step of the current South African multi-visit standard of care, in which women undergo cytology screening and are required to return to the clinic multiple times for results, triage, and pre-cancer treatment, if necessary. First, widespread implementation of cytology and triage demands critical infrastructure, equipment, and adequately trained personnel in clinics and laboratories, all of which are lean in-country in the public-sector healthcare network (1, 2, 21, 22). Meeting supply and cold chain requirements for cryotherapy treatment of cervical lesions proves challenging (23), and the need for multiple clinic visits results in notable loss to follow-up (23, 24). These barriers emphasize the imperative for more efficient and less resource-intensive screening strategies such as single-visit screening and treatment approaches that employ high performance technologies like HPV DNA testing and genotyping.

A multi-pronged approach and scale-up of appropriate interventions is needed to reach WHO 90-70-90 cervical cancer elimination goals. However, prevention and management of cervical cancer are associated with considerable clinical and economic costs with implications for accessing effective care in LMICs (25–28). The interaction between HIV and HPV compounds the health and economic burden and underscores the urgent need for prevention and early intervention strategies. We aimed to estimate the potential health outcomes, economic costs, and cost-effectiveness of single-visit cervical cancer screening strategies among women in KwaZulu-Natal, South Africa.

## 2 Materials and methods

### 2.1 Overview

To project future outcomes of multiple cervical cancer screening interventions, we utilized the Data-driven Recommendations for Interventions against Viral InfEction (DRIVE) model, which simulates HPV and HIV transmission, co-infection, and natural history. Output from the DRIVE model were used to estimate future costs associated with screening, testing, and treatment. Health and economic outcomes were jointly evaluated to assess cost-effectiveness.

### 2.2 Transmission model

The DRIVE model is a compartmental model that has been calibrated and described in previous publications (29, 30). The model simulates an open population of men and women aged 0-79 years, stratified by sex, 5-year age group, sexual risk group, and HIV- and HPV- associated health states. HIV health states are stratified by CD4 cell count and viral load (**Supplementary Figure S3**), while HPV health states are stratified by pre-cancerous lesions and stages of cervical cancer (**Supplementary Figure S2**). The model simulates demographic dynamics; heterosexual transmission of oncogenic HPV and HIV infection; HIV-related interventions such as ART, voluntary male medical circumcision, and condoms; HPV vaccination; natural history of HPV infections; and cervical cancer screening, diagnosis, treatment, and mortality. The model represents interactions between HIV and HPV, in that HPV acquisition and progression risks increase with declining CD4 count among individuals with untreated HIV, and screening and treatment performance vary by HIV status.

Model dynamics are governed by a system of ordinary differential equations solved in MATLAB (version R2022a) using a 4^th^ order Runge-Kutta numerical method. HPV is introduced in 1925 to allow HPV transmission dynamics and cervical cancer incidence to equilibrate prior to the introduction of HIV infection in 1980. At each 2-month time step, differential equations were evaluated to estimate population demographics and the number of persons in each infection, disease, or treatment state for the following time step. The dynamic nature of the model captured population-level effects such as herd immunity. Description of model processes, calibration, parameters, and data sources are in the **Supplementary Material** and previous publications (29, 30).

### 2.3 Strategies and scenarios

We used the 25 best-fitting parameter sets from model calibration to simulate seven primary scenarios (**Table 1**). We evaluated the status quo and six comparator strategies with nonavalent HPV (9vHPV) vaccine coverage and varying screening modalities, frequencies, and loss to follow-up between screening and treatments. In the status quo scenario, we simulated one-time screening between the ages of 35 and 39 with a multi-visit screening and treatment strategy and 57% 9vHPV vaccine coverage, based on a 2020 observation (31). The multi-visit strategy reflects the current South African standard of care of cytology screening, triage with colposcopy, and treatment with cryotherapy or large loop excision of the transformation zone (LLETZ). The need for multiple visits results in an estimated 64% of screen-positive women who are lost to follow-up for treatment (23, 24, 32).

**Table 1:** Primary screening strategies and scenarios.

Scenario		Number of visits for screening and treatment	Loss to follow-up between screening to treatment	Screening strategy	Screening frequency
1	Status quo	Multi-visit	28% for colposcopy; 50% for cryotherapy or LLETZ	Cytology + colposcopy triage	One-time^1^
2	Comparators	Single-visit	5% for thermal ablation; 20% for LLETZ	HPV DNA testing	One-time^1^
3					Repeat^2^
4				HPV DNA genotyping	One-time^1^
5					Repeat^2^
6				HPV DNA testing + AVE triage	One-time^1^
7					Repeat^2^

AVE, automated visual evaluation; HIV, human immunodeficiency virus; HPV, human papillomavirus; LLETZ, large loop excision of the transformation zone.

^1^Once-per-lifetime screening between ages 35 to 39.

^2^Repeated screening every 5 years for women living with HIV and twice-per-lifetime for women without HIV.

Our comparator scenarios assumed sustained 57% coverage of 9vHPV and a switch to single-visit strategies, where both screening and pre-cancer treatment occur during the same visit. In these single-visit scenarios, we assumed lower loss to follow-up compared to the status quo, with rates reduced to 5% for thermal ablation and 20% for LLETZ. We evaluated three screening strategies: 1) HPV DNA testing, 2) HPV DNA testing with genotyping, and 3) HPV DNA testing and triage with automated visual evaluation (AVE), a new machine learning-based technology with demonstrated high performance (33–36). Each strategy was implemented either: 1) once-per-lifetime between ages 35 to 39 years for all women (“one-time” screening) or 2) twice-per-lifetime between ages 35 to 39 and 45 to 49 for women without HIV and every 5 years for WLHIV, starting from age 25 (“repeat” screening).

### 2.4 Outcomes

Model outcomes included cervical cancer cases and deaths averted, life-years saved, and disability-adjusted life-years (DALYs) averted. Disability weights for cervical cancer health states were derived from Global Burden of Disease (**Table 2**) (48). We adopted the South African Ministry of Health perspective for costs, encompassing direct medical expenses. Aggregated costs of cervical cancer screening, triage, and the treatment and care of pre-cancer, cervical cancer, and HIV were derived from published studies (37–39, 41, 43–47, 49–51). HPV vaccination costs accounted for the 9vHPV vaccine product, with an additional 5% for wastage, 4.5% for transportation and handling, and 15% for distribution and delivery, based on prior studies (43, 44, 50, 51). Costs were converted and inflated to 2022 US dollars. Costs and outcomes were projected over lifetime time horizon of 100 years from 2023 to 2122 to capture the full impacts of the interventions and were discounted at a rate of 3% per year (52, 53). We reported our results according to HPV-FRAME, a consensus statement and quality framework for modelled evaluations of HPV prevention, and Consolidated Health Economic Evaluation Reporting Standards (CHEERS) 2022, the guidance for reporting health economic evaluations (54, 55) (**Supplementary Sections VII.a** and **VII.b**, respectively).

**Table 2:** Key cost-effectiveness analysis inputs.

Parameter	Estimate (Range)	Source
Costs (2022 USD)		
Screening & Triage		
Cytology	$12.78 ($10.22 – $17.85)	(37, 38)
Colposcopy	$105.91 ($84.73 – $127.09)	(37)
HPV DNA testing	$47.17 ($37.74 – $56.60)	(37, 39)
HPV DNA genotyping	$93.96 ($75.17 – $112.75)	(37, 39)
AVE	$5.74 ($4.59 – $6.89)	Assumption based on (37)
Pre-cancer Treatment		
Cryotherapy	$5.95 ($4.76 – $7.14)	(40)
LLETZ	$76.28 ($52.15 – $206.25)	(38, 40, 41)
Thermal ablation	$10.02 ($8.02 – $12.02)	(41, 42)
HPV Vaccination		
Nonavalent vaccine cost (per dose)	$106.98 ($85.58 – $128.38)	In-country source (43, 44)
Cervical Cancer		
Staging	$293.91 ($235.13 – $352.69)	(45)
Hysterectomy - radical	$1,829.23 ($1,463.38 – $2,195.08)	(45)
Local cervical cancer	$10,795.77 ($8,636.62 – $12,954.92)	(45)
Regional cervical cancer	$10,795.77 ($8,636.62 – $12,954.92)	(45)
Distant cervical cancer	$10,763.99 ($8,611.19 – $12,916.79)	(45)
HIV		
On ART, virally suppressed	$52.23 ($41.78 – $62.68)	(46)
CD4 > 500	$37.32 ($26.13 – $44.78)	(46, 47)
CD4 350 - 500	$39.57 ($31.66 – $47.48)	(47)
CD4 200 - 350	$39.98 ($31.98 – $47.98)	(47)
CD4 <=200	$98.89 ($79.11 – $118.67)	(47)
Additional Inputs		
Disability weights		
Local cervical cancer	0.288 (0.193-0.399)	(48) (proxy: diagnosed cancer and primary therapy)
Regional cervical cancer	0.451 (0.307-0.6)	(48) (proxy: metastatic cancer)
Distant cervical cancer	0.54 (0.377-0.687)	(48) (proxy: terminal phase)
Hysterectomy	0.049 (0.031-0.072)	(48) (proxy: controlled phase)
HPV vaccination		
Number of doses	2 (1 – 3)	Assumed
Additional cost for waste (% of vaccine product cost)	5% (4% – 6%)	(49)
Additional cost for transportation and handling (% of vaccine product cost)	4.5% (2% – 7%)	(50)
Additional cost for delivery/distribution (% of vaccine product cost)	15% (10% – 20%)	(44, 51)

AVE, automated visual evaluation; HIV, human immunodeficiency virus; HPV, human papillomavirus; LLETZ, large loop excision of the transformation zone; USD, US dollars.

### 2.5 Statistical analysis

The comparative performance of each scenario was evaluated using the incremental cost effectiveness ratio (ICER), computed as the additional cost divided by the additional health benefit (in DALYs) of one strategy compared with the next less costly strategy. Strategies that were more costly and less effective than an alternative (strongly dominated) or had higher ICERs compared to a more effective alternative (weakly or extended dominated) were considered inefficient and removed from the calculations in that analysis following standard practice. For all non-dominated scenarios, we report the median ICER from simulations using the 25 best-fitting parameter sets, along with the minimum and maximum values. We adopted a commonly utilized willingness to pay threshold (or cost-effectiveness threshold) of South Africa’s gross domestic product (GDP; $6,776 per capita in 2022) to determine the most optimal strategy (56). However, given the lack of consensus on which thresholds are most appropriate in LMICs, we applied several additional thresholds ranging from $2,221 to $8,909 based on health opportunity costs ($2,221 and $8,909) and 50% of the GDP per capita ($3,388) (57, 58). The cost-effectiveness analyses were conducted using R (version 4.2.1).

### 2.6 Sensitivity analysis

We conducted one-way sensitivity analyses of costs and disability weights, informed by published literature or by adjusting by 20% when no data were available. Additional scenario analyses were conducted in which we introduced: a more optimistic estimate of 90% 9vHPV vaccine coverage, increased loss to follow-up in single-visit strategies (30% for thermal ablation and 50% for LLETZ), and a shortened time horizon of 50 years. We also explored the impact of AVE as a primary screening strategy 1) at optimal test performance and 2) with 20% reduction in test sensitivity and specificity.

## 3 Results

Clinical and economic outcomes for our primary scenarios are summarized in **Table 3** and **Supplementary Table S34**. Over the 100-year time horizon, the status quo scenario was estimated to result in 69,294 cervical cancer cases, 43,950 cervical cancer-related deaths, and 188.13 million life-years. All comparator scenarios in the base and sensitivity analyses demonstrated improved health outcomes and were therefore more effective than the status quo. Relative to the status quo, repeat screening achieved lower cervical cancer incidence (29.5% to 37.1% reduction) and mortality (25.8% to 35.1% reduction) compared to one-time screening (7.1% to 9.4% and 6.0% to 9.0%, respectively). Further, repeat screening with HPV DNA testing was associated greater reduction in cervical cancer cases (37.1%) and mortality (35.1%) compared to HPV DNA genotyping (29.5% and 25.8%, respectively) and HPV DNA testing with AVE triage (32.6% and 31.2%, respectively).

**Table 3:** Health and cost impact of cervical cancer screening strategies in South Africa^1^.

Description	CC Cases Averted, % Change^2^	CC Deaths Averted, % Change^2^	Total Costs, 2022 USD	DALY Averted, Count	ICER^3^, $ per DALY averted
Status quo^4^	–	–	12.786 B (11.25 B-13.78 B)	–	–
One-time HPV DNA testing^5^	9.4 (7.9-11.1)	9.0 (7.5-10.5)	12.83 B (11.30 B-13.82 B)	34,080 (14,221-112,877)	Dominated
One-time HPV DNA genotyping^5^	7.1 (5.5-8.4)	6.0 (4.5-7.5)	12.89 B (11.37 B-13.88 B)	25,657 (10,044-85,820)	1,398 (442-3,478)
One-time HPV DNA testing with AVE triage^5^	7.9 (6.3-9.6)	7.4 (6.2-9.1)	12.83 B (11.30 B-13.82 B)	26,387 (11,717-95,033)	Dominated
Repeat HPV DNA testing^6^	37.1 (30.7-41.7)	35.1 (29.2-39.5)	13.00 B (11.47 B-13.99 B)	91,590 (37,522-254,599)	3,194 (1,488-7,599)
Repeat HPV DNA genotyping^6^	29.5 (25.6-36.7)	25.8 (22.8-33.1)	13.19 B (11.67 B-14.19 B)	68,623 (29,258-203,559)	Dominated
Repeat HPV DNA testing with AVE triage^6^	32.6 (27.1-38.1)	31.2 (25.9-35.6)	13.00 B (11.47 B-13.99 B)	75,615 (32,214-226,500)	Dominated

%, Percent; AVE, automated visual evaluation; B, billion; CC, cervical cancer; DALY, disability-adjusted life-year; HIV, human immunodeficiency virus; HPV, human papillomavirus; ICER, incremental cost-effectiveness ratio; USD, US dollar.

^1^We report the median estimates across the 25 best fitting parameter sets, along with the minimum and maximum in parentheses. All costs and outcomes were discounted at 3% annually. ^2^Reflects the percent reduction in CC cases and CC-related deaths compared to the status quo. ^3^ICER is reported for nondominated strategies. Dominated strategies, which exhibited higher costs and lower effectiveness than an alternative or higher ICERs compared to a more effective alternative, were deemed inefficient. ^4^Multi-visit screening and treatment strategy between ages 35 to 39. ^5^Single-visit screening and treatment strategy once per lifetime between ages 35 to 39. ^6^Single-visit screening and treatment strategy every 5 years for women living with HIV and twice at ages 35-39 and 45-49 for women without HIV for women without HIV.

**Figure 1:**
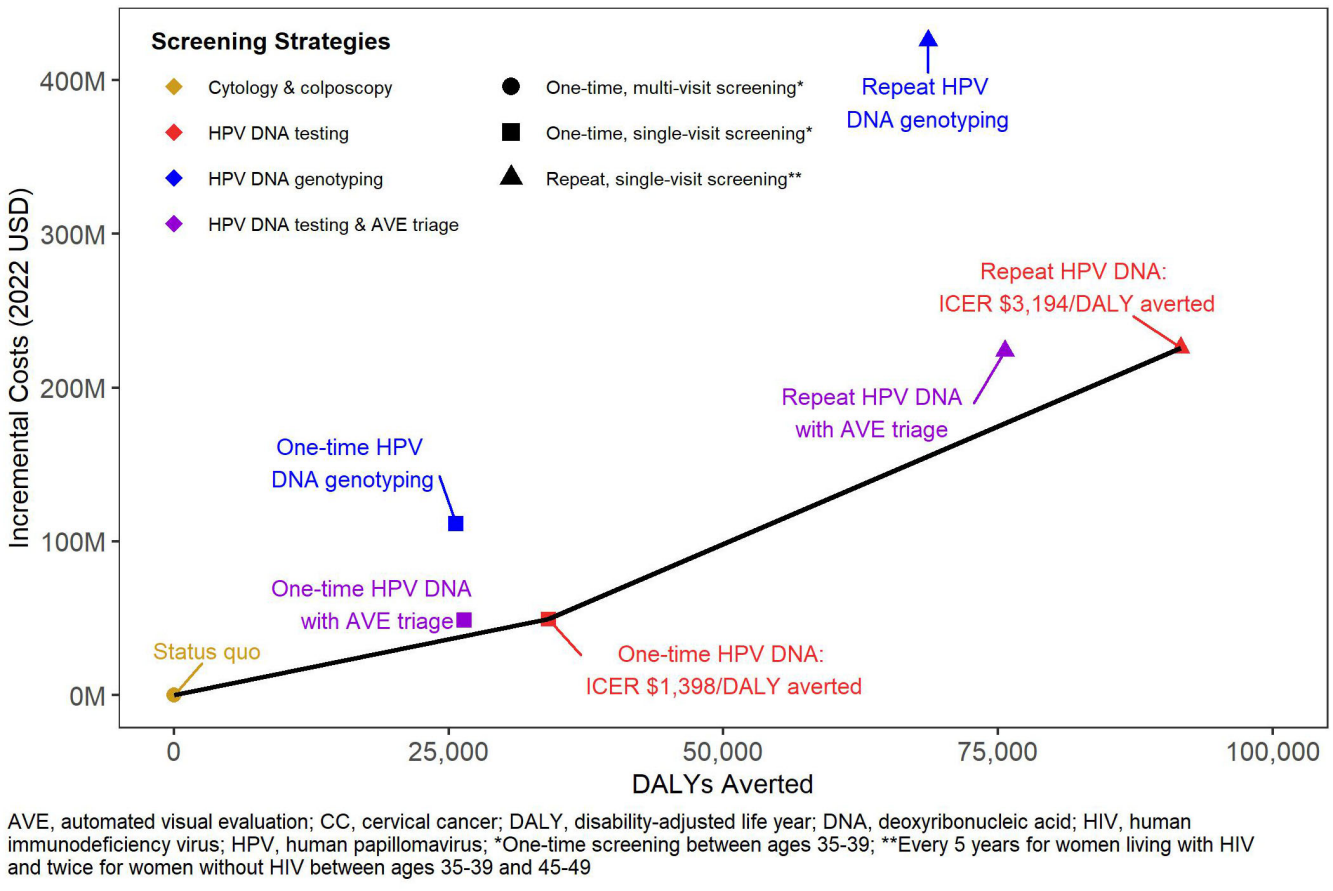
Cost-effectiveness of primary cervical cancer screening strategies.

The status quo screening scenario was associated with $12.79 billion in direct medical costs over the next 100 years (**Table 3**). Among the single-visit strategies, we found greater increases in costs of repeat screening (1.8% to 3.3%) compared to one-time screening (0.4% to 0.9%) across all technologies. HPV DNA testing with genotyping was more costly than HPV DNA testing alone and with AVE triage. **Figure 1** shows the efficiency frontier with the incremental costs and DALYs for each scenario compared to the status quo. Repeat screening with HPV DNA testing was the most effective strategy below the willingness to pay threshold of South Africa’s GDP per capita (ICER: $3,194 per DALY averted). However, when we assumed the lowest bound threshold of $2,221, one-time screening with HPV DNA testing became the optimal strategy (ICER: $1,398 per DALY averted).

**Figure 2:**
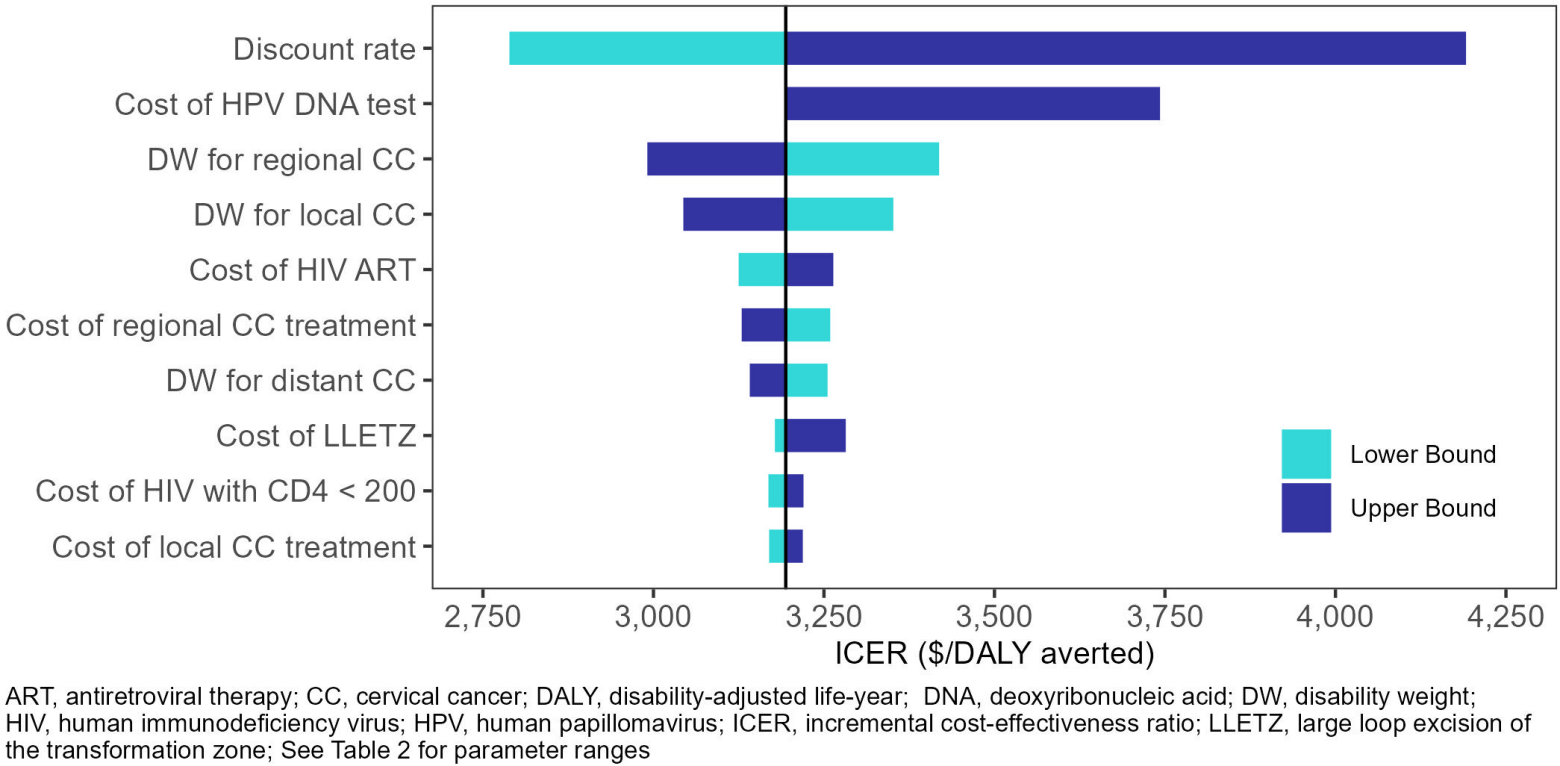
One-way sensitivity analysis – Repeat single-visit screening with HPV DNA testing.

**Figure 3:**
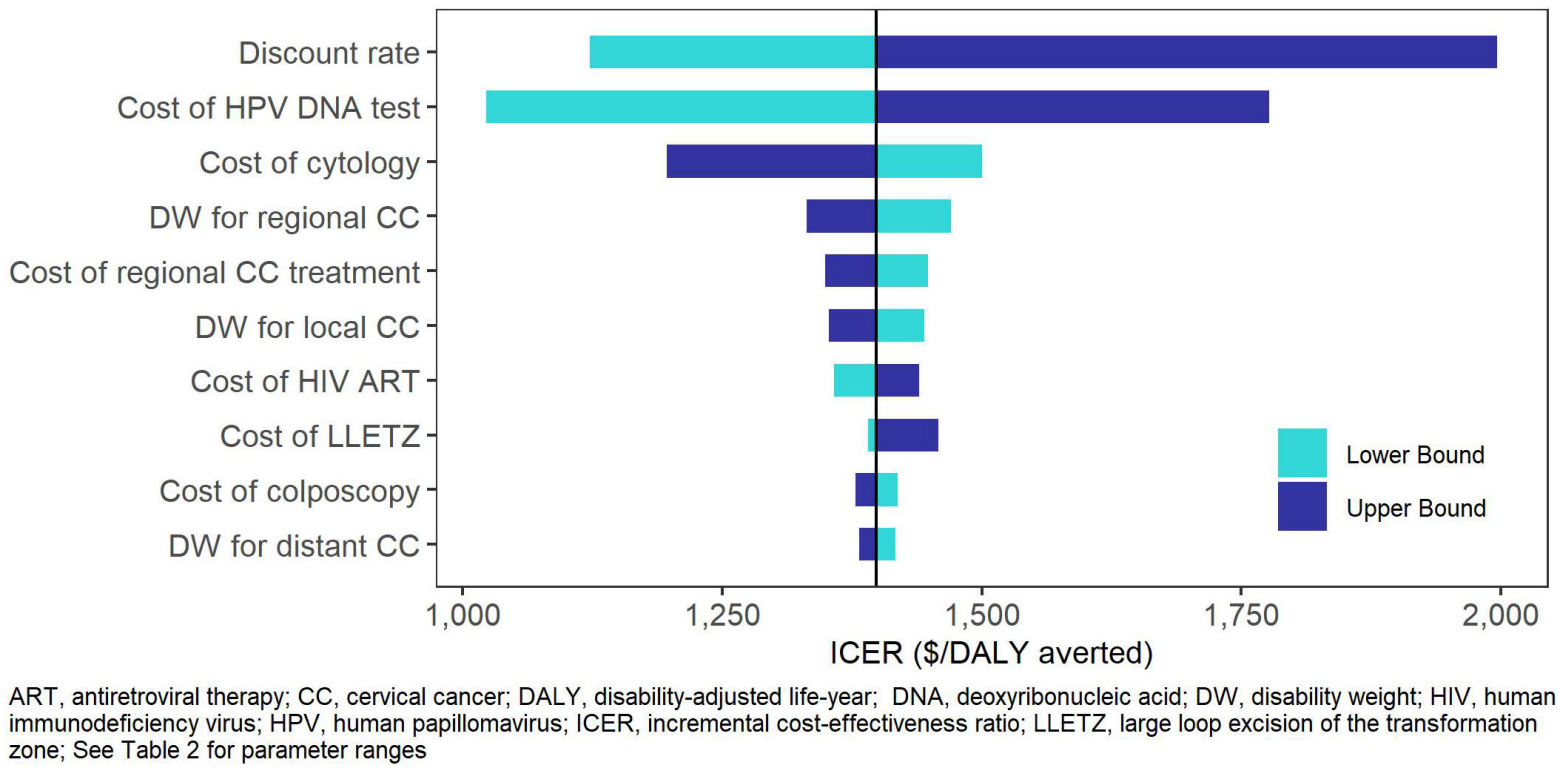
One-way sensitivity analysis – One-time single-visit screening with HPV DNA testing.

In the one-way sensitivity analyses (**Figures 2**, **3**), the parameters with the greatest impact on both ICERs were the discount rate and cost of HPV DNA testing. In the scenario analyses (**Table 4**, **Supplementary Table S35**), increasing 9vHPV vaccine coverage to 90% with single-visit screening and treatment strategies had notable impact on cervical cancer outcomes, averting up to 44.3% of cervical cancer cases and 41.2% of deaths, and increasing costs up to 6.3%. At our base willingness to pay threshold, the optimal strategy remained repeat screening with HPV DNA testing when assumptions of vaccine coverage and loss to follow-up were increased and when the time horizon was shortened to 50 years, but AVE became optimal when we assumed its use as a primary screening strategy.

**Table 4:** Optimal screening strategy under different assumptions across varying willingness to pay thresholds.

	Base value	Willingness to Pay Threshold			
		$2,221	$3,388 (50% of GDP)	$6,776 (GDP)	$8,909
Primary scenarios	––	One-time HPV DNA testing: $1,398/DALY averted	Repeat DNA testing: $3,194/DALY averted	Repeat DNA testing: $3,194/DALY averted	Repeat DNA testing: $3,194/DALY averted
LTFU increased to 30% for TA/50% for LLETZ	5%/20%	Dominated	One-time HPV DNA testing: $2,193/DALY averted	Repeat DNA testing: $4,134/DALY averted	Repeat DNA testing: $4,134/DALY averted
Time horizon of 50 years	100	One-time HPV DNA testing: $1,615/DALY averted	Repeat DNA testing: $3,326/DALY averted	Repeat DNA testing: $3,326/DALY averted	Repeat DNA testing: $3,326/DALY averted
AVE for primary screening	Not included	Repeat AVE: $915/DALY averted	Repeat AVE: $915/DALY averted	Repeat AVE: $915/DALY averted	Repeat AVE: $915/DALY averted
AVE for primary screening with 20% lower sensitivity and specificity	Not included	Repeat AVE: $984/DALY averted	Repeat AVE: $984/DALY averted	Repeat AVE: $984/DALY averted	Repeat AVE: $984/DALY averted
90% HPV vaccine coverage	57%	Dominated	Dominated	Repeat DNA testing: $4,605/DALY averted	Repeat DNA testing: $4,605/DALY averted
90% HPV vaccine coverage with increased LTFU: 30% for TA/50% for LLETZ	57%/5%/20%	Dominated	Dominated	Repeat DNA testing: $5,740/DALY averted	Repeat DNA testing: $5,740/DALY averted
90% HPV vaccine coverage with AVE for primary screening	57%/Not included	Dominated	Repeat AVE: $3,222/DALY averted	Repeat AVE: $3,222/DALY averted	Repeat AVE: $3,222/DALY averted
90% HPV vaccine coverage with AVE for primary screening with 20% lower sensitivity and specificity	57%/Not included	Dominated	Dominated	Repeat AVE: $3,554/DALY averted	Repeat AVE: $3,554/DALY averted

AVE, automated visual evaluation; DALY, disability-adjusted life-year; GDP, gross domestic product; HIV, human immunodeficiency virus; HPV, human papillomavirus; LLETZ, large loop excision of the transformation zone; USD, US dollar.

The strategies listed were the most effective under the specified willingness to pay threshold among non-dominated strategies. These strategies were not dominated in 100% of our 25 best-fitting parameter sets, and the ICERs listed are the median values.

## 4 Discussion

Our paper contributes to the limited literature evaluating the economic and clinical benefits of cervical cancer screening interventions, while accounting for the impact of HIV (59). To our knowledge, this is the first cost-effectiveness analysis in the South African context to incorporate DALYs averted as part of the cost-effectiveness measure evaluating cervical cancer screening and interventions. The use of a standardized outcome such as DALYs allows policy and decision makers to weigh costs and outcomes across disease states and interventions. Given funding and resource constraints in LMICs, implementing cost-effective cervical cancer prevention strategies is imperative to achieving WHO 90-70-90 cervical cancer elimination goals.

We found that repeat single-visit screening with HPV DNA testing was the most effective strategy under our willingness to pay threshold; one-time single-visit screening with HPV DNA testing also had an ICER under our threshold but was less effective than repeat screening. Although more frequent screening was associated with increased costs, our model substantiates its added clinical benefits of reduced cervical cancer incidence, mortality, and DALYs, and its cost-effectiveness, particularly among WLHIV, as recommended by WHO (60). Previous studies found that same-day screening and treatment could improve cervical cancer screening uptake and reduce the burden in South Africa (29, 61), and our findings suggest that implementing single-visit strategies could yield greatly improved health outcomes at comparatively modest increases in costs.

We demonstrate both the effectiveness and cost-effectiveness of screening with HPV DNA testing, further supporting WHO cervical cancer screening recommendations (60). However, as evident by prior studies and our analysis, HPV DNA testing is associated with higher costs (0.4% and 1.8% increase with one-time and repeat screening, respectively) (37, 38, 45, 62), and real-world implementation and public sector scale-up of HPV DNA testing in KwaZulu-Natal will require substantial financial investment, resources, and time. Drivers of these additional costs may be attributed to more women receiving pre-cancer treatment because of HPV DNA’s higher test sensitivity and lower loss to follow-up from the single-visit strategies, but it is also noted that costs may be offset by averting cervical cancer cases and the need for cervical cancer treatment.

Our findings are consistent with several economic evaluations that have demonstrated the cost-effectiveness of single-visit screening and treatment, HPV DNA testing, and HPV vaccination in Sub-Saharan Africa (44, 45, 62–65). For example, a prior study by Zimmermann et al. found that the cost of single-visit screening strategies at an HIV clinic in Kenya was lower than two-visit strategies, and HPV DNA testing was the most effective strategy when screening and treatment were provided in a single visit (66). Conversely, alternative strategies such as HPV genotyping and visual inspection with acetic acid may be optimal in other contexts (37, 67–70). Lew et al. identified repeat HPV screening with partial genotyping to be the optimal and cost-saving strategy in New Zealand (67), highlighting the potential benefits of newer technologies while emphasizing the importance of repeat screening. However, when comparing strategies and economic evaluations across resource settings, it is important to consider differences in the burdens of cervical cancer and HIV as well as barriers such as limited infrastructure, resources, and trained personnel (19, 71)

Given the interaction of HPV and HIV, mathematical models have also been used in numerous studies to evaluate cervical cancer interventions among WLHIV (59), and our results align with previous cost-effectiveness studies that modeled coinfection in South Africa (37, 45, 72–75). Similar to our findings, Campos et al. and Goldie et al. concluded that HPV DNA test and treat was the most cost-effective strategy (45, 76). In contrast, Lince-Deroche et al. found visual inspection with acetic acid to be most cost-effective, attributing the increased colposcopy triage costs to HPV DNA testing’s higher sensitivity and lower specificity (37); however, their analysis focused on programmatic screening and triage costs and did not account for costs of pre-cancer treatment, cervical cancer, and cervical cancer treatment. While visual inspection with acetic acid may demonstrate short-term cost-effectiveness, our study highlights the importance of incorporating downstream costs and benefits and suggests that HPV DNA testing would be cost-effective long-term. Our findings build upon these prior economic analyses by emphasizing the cost-effectiveness of single-visit screening with HPV DNA testing in South Africa and highlighting the benefits of more frequent screening, particularly among WLHIV.

Our results were sensitive to assumptions about loss to follow-up. In our primary scenarios (base case), we assumed a loss to follow-up rate of 5% for thermal ablation treatment and 20% for LLETZ. In sensitivity analyses, we applied more conservative estimates, increasing loss to follow-up to 30% and 50%, respectively. Despite the higher ICERs with increased loss to follow-up, repeat screening with HPV DNA testing persisted as the most effective strategy under the base willingness to pay threshold.

To assess the potential impact of scaling 9vHPV vaccination, we considered a more optimistic vaccine coverage of 90%. Our findings suggest that vaccine scale-up would prevent substantially more cervical cancer cases and cervical cancer-related deaths, and repeat HPV DNA testing remained the optimal screening strategy. It is important to note that the 9vHPV vaccine in our model covers nine HPV types compared to two and four types in the bivalent and quadrivalent HPV vaccine, respectively, but the 9vHPV vaccine has not been widely rolled out in South Africa. The cost of the 9vHPV vaccine can be up to 20 times more expensive than the bi- and quadrivalent alternatives and the costs of vaccine delivery may be lower than estimated in our model (44, 77, 78). Our assumption of switching to 9vHPV vaccine coverage may overestimate the effectiveness and cost of HPV vaccination and, consequently, diminish the estimated health impact and cost-effectiveness of cervical cancer screening and treatment strategies, which address the residual burden of cases not prevented by vaccination. Therefore, our ICERs are conservative.

Our choice of willingness to pay thresholds and discount rate had notable impact on our conclusions of cost-effectiveness. We find repeat single-visit screening with HPV DNA testing is the most optimal strategy at all study thresholds equal to or higher than 50% of GDP per capita ($3,388). However, one-time HPV DNA testing was the most effective strategy at our lowest threshold of $2,221, emphasizing how recommendations and decisions may differ depending on the willingness to pay threshold employed by policy makers. Further, applying a higher discount rate of 6% yield an ICER exceeding $3,388 for repeat HPV DNA testing, and the strategy would no longer be deemed cost-effective.

We employed a 100-year lifetime time horizon to capture the full health and economic impact of the interventions simulated. However, because longer time horizons inherently introduce greater uncertainty, the projected long-term health and economic outcomes may be less reliable. We conducted a sensitivity analysis using a 50-year time horizon, and our conclusions remained consistent, with repeat screening with HPV DNA testing emerging as the most effective strategy under the cost-effectiveness threshold.

Our analysis highlights potential cost-effective opportunities for recent innovations with high sensitivity and specificity such as HPV genotyping and AVE. Although our findings demonstrate the clinical benefits of HPV DNA genotyping, the ICER exceeded our threshold, likely due to the increased costs from testing and treatment, but newer technologies for genotyping have the potential to lower testing prices. Moreover, while AVE was not cost-effective for triage in our two primary scenarios, it became the optimal strategy when we assumed its use as a primary screening strategy, highlighting its potential future role in cervical cancer screening. However, it should be noted that the costs and performance of AVE are currently highly uncertain, and additional data will be needed to generate more reliable cost-effectiveness estimates.

A key strength of this analysis is the use of a dynamic HIV-HPV transmission model, allowing us to simulate the natural history of HIV, HPV, and cervical cancer, along with their interaction and transmission. We also assessed numerous strategies ranging from current South African standards (cytology with colposcopy), single-visit screening and treatment approaches, WHO’s current recommendations (HPV DNA testing with and without genotyping), scaled 9vHPV vaccine coverage, and a promising novel technology leveraging machine learning (AVE).

Our study is subject to several limitations. First, we use the Ministry of Health perspective and do not include societal costs such as productivity and travel time costs, which would likely increase our ICERs if the societal costs associated with the screening and treatment strategies are substantial. However, this approach may also have the potential to decrease ICERs if averting cervical cancer and death would have profound improvement on productivity costs. Further, we did not collect primary cost data but rather derived our cost-estimates from published costing studies and input from in-country experts in South Africa. Lastly, when calculating DALYs averted, we included only disability from cervical cancer because disability weights for coinfection of HIV and HPV/cervical cancer have not yet been estimated. We considered the quality-of-life impacts for cervical cancer to be of greater interest since our interventions focused on cervical cancer prevention.

In conclusion, we find that adopting single-visit strategies with high performance HPV DNA testing will improve the impact of cervical cancer prevention resources. In KwaZulu-Natal and similar LMIC settings with high HIV prevalence, repeat screening every five years for WLHIV and twice between ages 35 to 39 and 45 to 49 for women without HIV would be the optimal cervical cancer screening and treatment approach. Our findings can inform resource allocation and policy deliberations regarding optimal strategies to reach the WHO 90-70-90 cervical cancer elimination goals by 2030.

## Data availability statement

The raw data supporting the conclusions of this article will be made available by the authors, without undue reservation.

## Author contributions

JT: Conceptualization, Formal analysis, Methodology, Software, Visualization, Writing – original draft, Writing – review & editing. CH: Data curation, Formal analysis, Methodology, Software, Writing – review & editing. CB: Data curation, Formal analysis, Methodology, Software, Writing – review & editing. TP-P: Resources, Writing – review & editing. RB: Conceptualization, Methodology, Supervision, Writing – review & editing, Funding acquisition. DR: Conceptualization, Methodology, Supervision, Writing – review & editing, Funding acquisition. MS: Methodology, Supervision, Writing – review & editing, Funding acquisition.
